# Comparison and impact of associated anomalies on the anal position index in neonates with anorectal malformation

**DOI:** 10.1186/s13104-022-06186-x

**Published:** 2022-09-07

**Authors:** Ririd Tri Pitaka, Aditya Rifqi Fauzi, Akhmad Makhmudi, Gunadi

**Affiliations:** Pediatric Surgery Division, Department of Surgery, Faculty of Medicine, Public Health and Nursing, Universitas Gadjah Mada/Dr. Sardjito Hospital, Jl. Kesehatan No. 1, Yogyakarta, 55281 Indonesia

**Keywords:** Anal position index, Anorectal malformation, Associated anomalies, Neonates

## Abstract

**Objective:**

Some prognostic factors have affected the functional outcomes of patients with anorectal malformations (ARM) after definitive surgery, including the associated anomalies. Moreover, the anal position index (API) study in neonates from developing countries is minimal. We aimed to (1) compare the API between neonates with ARM and controls; and (2) determine the impact of associated anomalies on the API in neonates with ARM.

**Results:**

We ascertained 68 subjects: 35 neonates with ARM and 33 controls. The API of neonates with ARM was similar to controls, either male or female neonates (*p* = 0.51 and 0.90, respectively). Interestingly, the API in ARM males with associated anomalies (0.42 ± 0.07) was significantly lower than in control males (0.48 ± 0.02) (*p* = 0.005). Moreover, the API of ARM neonates with vertebral anomalies (0.35 ± 0.04) was lower than ARM neonates without vertebral anomalies (0.47 ± 0.07) (*p* = 0.021). In conclusion, associated anomalies and sex might affect the API in neonates with ARM. These findings should be considered and informed during counseling to the parents regarding the prognosis of functional outcomes in ARM neonates, particularly with associated anomalies.

**Supplementary Information:**

The online version contains supplementary material available at 10.1186/s13104-022-06186-x.

## Introduction

Anal position index (API) is a quantitative measurement to determine the normal anal position by defining the ratio of scrotum–anal distance to scrotum-coccyx distance and fourchette-anal distance to fourchette–coccyx distance for males and females, respectively [1].

Some prognostic factors have affected the functional outcomes of patients with anorectal malformations (ARM) after definitive surgery, including the associated anomalies [2–5]. The associated anomalies found in ARM are genitourinary (40–50%), followed by cardiovascular (30–35%), spinal cord tethering (25–30%), gastrointestinal anomalies (5–10%), and VACTERL (4–9%) anomalies [2]. Moreover, the API study in neonates from developing countries is minimal [6, 7]. Here, we aimed: (1) to compare the API between neonates with ARM and controls; and (2) to determine the impact of associated anomalies on the API in neonates with ARM.

## Main text

### Material and methods

#### Patient samples

We evaluated the medical records of neonates with ARM and controls at our institution from November 2018 and April 2021. Neonates with incomplete medical records were excluded. The Institutional Review Board of the Faculty of Medicine, Public Health, and Nursing, Universitas Gadjah Mada/Dr. Sardjito Hospital (KE/FK/1191/EC/2020) approved this study. Written informed consent was obtained from the parents or guardians of neonates.

### Anal position index (API)

For controls, API was determined as the ratio of scrotum–anal distance to scrotum-coccyx distance and of fourchette-anal distance to fourchette–coccyx distance for males and females, respectively. For neonates with ARM, API was defined as the ratio of scrotum—the center of anal dimple distance to scrotum-coccyx distance and fourchette-center of anal dimple distance to fourchette–coccyx distance for males and females, respectively (Additional file 1: Fig. S1). API was determined prior to surgery at the time of identification of an ARM at birth. Anal dimple is an area of usually elevated, hyperpigmented skin in the perineum midline in neonates with ARM. However, its epicenter might be depressed (anal fossette) [8].

### Statistical analysis

The data were presented as frequency (percentage) and mean ± SD. The significance of mean differences among groups was determined using the One-way Anova test. The *p*-value of < 0.05 was considered statistically significant. The IBM Statistical Package for Social Science (SPSS) version 21 (IBM Corp., Chicago) was used to perform all statistical analyses.

## Results

### Baseline characteristics of neonates

We ascertained 68 neonates: 35 neonates with ARM, consisting of 30 males and five females, and 33 controls, involving 17 males and 16 females. Most associated anomalies in ARM neonates were congenital heart disorder (63.6%), followed by Down syndrome (54.5%), vertebral anomaly (27.3%), trachea-oesophageal anomaly (27.3%), and limb anomaly (18.2%). The frequency of associated anomalies was a significant difference between male and female neonates (*p* = 0.005) (Additional file 2: Table S1) [9].

### Comparison of API between neonates with ARM and controls

The API of neonates with ARM was similar to controls, either male or female neonates (*p* = 0.51 and 0.90, respectively) (Table 1) [9].

**Table 1:** Comparison of API between neonates with ARM and controls according to sex

	Controls (mean ± SD)	ARM (mean ± SD)	*p*-value
Sex			
Male	0.48 ± 0.02	0.47 ± 0.06	0.51
Female	0.35 ± 0.06	0.38 ± 0.11	0.90

*ARM* anorectal malformation, *SD* standard deviation

Next, we conducted a subgroup analysis of API differences of neonates with associated anomalies and controls stratified by subjects’ sex. (Table 2). The API of ARM males with associated anomalies (0.42 ± 0.07) was significantly lower than control males (0.48 ± 0.02) (*p* = 0.005) (Table 2).

**Table 2:** Comparison of API between ARM neonates with associated anomalies and controls stratified by subjects’ sex

	API (mean ± SD)	*p*-value
Male		
Controls	0.48 ± 0.02	Ref.
ARM without associated anomalies	0.49 ± 0.05	0.61
ARM with associated anomalies	0.42 ± 0.07	0.005*
Female		
Controls	0.35 ± 0.06	Ref.
ARM without associated anomalies	0.35 ± 0.01	0.73
ARM with associated anomalies	0.39 ± 0.16	0.91

*API* anal position index, *ARM* anorectal malformation, *SD* standard deviation

^*^p-value considered significant if *p* < 0.05

### Association of API and associated anomalies in neonates with ARM

The API of ARM neonates with vertebral anomalies (0.35 ± 0.04) was lower than ARM neonates without vertebral anomalies (0.47 ± 0.07) (*p* = 0.021), while the other associated anomalies were not correlated with the API (*p* > 0.05) (Table 3).

**Table 3:** Association of API and associated anomalies in neonates with ARM

Associated anomalies	API (mean ± SD)	*p*-value
Vertebral anomaly		
Yes	0.35 ± 0.04	0.021*
No	0.47 ± 0.07	
Heart anomaly		
Yes	0.42 ± 0.08	0.13
No	0.47 ± 0.08	
Trachea-esophageal anomaly		
Yes	0.45 ± 0.12	0.68
No	0.46 ± 0.08	
Limb anomaly		
Yes	0.42 ± 0.09	0.67
No	0.46 ± 0.08	
Down syndrome		
Yes	0.44 ± 0.08	0.44
No	0.46 ± 0.08	

*API* anal position index, *SD* standard deviation

^*^p-value considered significant if *p* < 0.05

## Discussion

Here, our study is able to provide new data on API in neonates, both controls and ARM from a developing country and different populations from previous reports [6, 7, 10–14]. We included the ARM patients with associated anomalies, including the vertebral anomaly, as the novelty of our study (vs*.* a normal sacrum was the inclusion criteria [12]). In addition, there are still variations in the API among studies [14]. These API variations might be due to different methods for API measurement and ethnic populations [15]. The measurement of API is suggested to determine the neonates' age to minimize the impact of ethnic variations on the API. However, a recent systematic review concluded that ethnic variations did not affect the API [8].

Our study shows that the API is affected by the associated anomalies in neonates with ARM, particularly in males. The associated anomalies have been associated with the functional outcomes of ARM patients after definitive surgery [2–5]. Therefore, it is suggested that a pediatric surgeon find any associated anomalies in neonates with ARM to ensure appropriate management and counseling for the parents [16].

Another novelty of our study is that we included all ARM neonates with and without associated anomaly *vs.* ARM neonates without sacral anomaly [12]. In addition, they suggested not using the anal dimple as the proposed neoanus since it might be anterior to the normal anus position [12]. A previous report also suggested that the API should not be used as the only parameter for surgical intervention [13] because the API measurement might be inaccurate, particularly in patients with constipation. Constipation might result in perineal elongation due to the fecal impaction in the rectum [8]. The association between API and constipation is controversial. While some studies noted their association [10, 16], other reports were not [7, 15, 18]. Notably, our study aimed to compare the API between neonates with ARM and controls and determine the impact of associated anomalies on the API in neonates with ARM. Therefore, we did not associate the API with ARM patients' prognosis after definitive surgery. Our study focused on the associated anomalies in ARM patients that affected the API.

Interestingly, our subgroup analysis revealed that the API in ARM males with associated anomalies was significantly lower than in control males (Table 2). Our study is the first report that analyzed the impact of sex and associated anomalies in ARM neonates on the API to the best of our knowledge. In addition, although it was not statistically significant, the ARM group with associated anomalies also had a lower birth weight. Therefore, studying the association between API and birth weight is interesting.

Among associated anomalies, only vertebral anomaly showed a significant association with the API, revealing that ARM neonates with vertebral anomaly have lower API than ARM neonates without vertebral anomaly (Table 3). These findings were also another novelty of our study. However, we did not determine the sacral ratio in our patients. It is known that patients with a lower sacral ratio may have an influence on the API. Furthermore, the most common associated anomalies in our patients were congenital heart disorder (63.6%), followed by Down syndrome (54.5%), vertebral (27.3%), trachea-oesophageal (27.3%), and limb anomaly (18.2%). In contrast, a previous study showed that the most common associated anomalies in ARM patients were genitourinary (39.7%), spinal anomaly (33.3%), and congenital heart disorder (16.1%) [19].

## Conclusions

Associated anomalies and sex might affect the API in neonates with ARM. These findings should be considered and informed during counseling to the parents regarding the prognosis of functional outcomes in ARM neonates, particularly with associated anomalies.

## Limitations

Several limitations of our study are as follows: (1) we included the neonates with ARM and controls from one institution only. It might not reflect all ethnic populations in Indonesia; (2) limited sample size. A multicentre study with a larger sample size is essential to clarify our findings; (3) we classified several vertebral anomalies within one group for analysis; (4) we did not associate the API and the location of the fistula. It is known that the higher the malformation, the closer the anal sphincter is located. Moreover, there are more associated anomalies in the ARM patients with a higher fistula location. These facts should be noted during the interpretation of our results.

## Supplementary Information

**Additional file1: Figure S1**. API measurement in controls: API was determined as the ratio of scrotum–anal distance to scrotum-coccyx distance and of fourchette-anal distance to fourchette–coccyx distance for males **a** and females **b**, respectively; and neonates with ARM: API was defined as the ratio of scrotum–center of anal dimple distance to scrotum-coccyx distance and fourchette-center of anal dimple distance to fourchette–coccyx distance for males **c** and females **d**, respectively.

**Additional file2: Table S1**. Baseline characteristics of neonates in our study.

## Data Availability

All data generated or analyzed during this study are included in the submission. The raw data are available from the corresponding author on reasonable request.

## Abbreviations

API: Anal position index
ARM: Anorectal malformation
SD: Standard deviation
