# Supplementary figures and images for: Comparison and impact of associated anomalies on the anal position index in neonates with anorectal malformation
Source: BMC Res Notes. 2022 Sep 7;15:294. doi: 10.1186/s13104-022-06186-x (PMC9450298; doi:10.1186/s13104-022-06186-x)

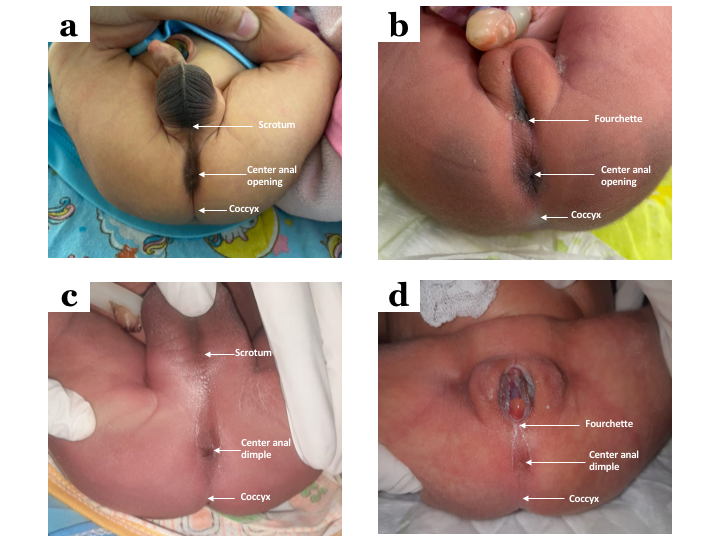

Supplement: Supplementary file 1 — Additional file1: Figure S1. API measurement in controls: API was determined as the ratio of scrotum–anal distance to scrotum-coccyx distance and of fourchette-anal distance to fourchette–coccyx distance for males a and females b, respectively; and neonates with ARM: API was defined as the ratio of scrotum–center of anal dimple distance to scrotum-coccyx distance and fourchette-center of anal dimple distance to fourchette–coccyx distance for males c and females d, respectively. [file 13104_2022_6186_MOESM1_ESM.tiff]
