# Supplementary material for: Comparison and impact of associated anomalies on the anal position index in neonates with anorectal malformation
Source: BMC Res Notes. 2022 Sep 7;15:294. doi: 10.1186/s13104-022-06186-x (PMC9450298; doi:10.1186/s13104-022-06186-x)
Supplement: Supplementary file 2 — Additional file2: Table S1. Baseline characteristics of neonates in our study. [file 13104_2022_6186_MOESM2_ESM.docx]

**Supplementary Table 1.** Baseline characteristics of neonates in our study.

| **Characteristic** | **Control**  **(N, %; mean ± SD)** | **ARM without associated anomalies (N, %; mean ± SD)** | **ARM with associated anomalies**  **(N, %; mean ± SD)** | ***p*-value** |
| --- | --- | --- | --- | --- |
| Sex   - Male - Female | 17 (51.5)  16 (48.5) | 22 (91.7)  2 (8.3) | 8 (72.7)  3 (27.3) | 0.005* |
| Age (days) | 1.3 ± 0.6 | 2.0 ± 1.2 | 2.1 ± 1.8 | 0.63 |
| Gestational age (weeks) | 37.7 ± 1.6 | 38.3 ± 2.6 | 38.4 ± 2.7 | 0.87 |
| Birthweight (gram) | 2964.7 ± 513.9 | 2756.7 ± 533.8 | 2618.2 ± 667.9 | 0.62 |
| Body length (cm) | 48.6 ± 1.9 | 47.7 ± 2.5 | 47.0 ± 2.1 | 0.20 |
| Associated anomalies   - Vertebral anomaly - Heart anomaly - Trachea-esophageal anomaly - Limb anomaly - Down syndrome |  |  | 3 (27.3)  7 (63.6)  3 (27.3)  2 (18.2)  6 (54.5) |  |

*, significant if *p*<0.05; ARM, anorectal malformation; cm, centimetre; SD, standard deviation
